# How Team Familiarity Mitigates Negative Consequences of Team Composition Disruptions: An Analysis of Premier League Teams

**DOI:** 10.1177/10596011231193176

**Published:** 2023-08-03

**Authors:** Surabhi Pasarakonda, Travis Maynard, Jan B. Schmutz, Patrick Lüthold, Gudela Grote

**Affiliations:** 1 27219 ETH Zurich, Zurich, Switzerland; 2 3447 Colorado State University, Fort Collins, CO, USA; 3 27217 University of Zurich, Zurich, Switzerland; 4 Lucerne, Department of Psychology, University of Fribourg, Switzerland

**Keywords:** team composition disruptions, team familiarity, team coordination, team performance, team dynamics

## Abstract

In today’s dynamic work environment, teams are increasingly confronted with disruptions. While there are different types of disruptions that teams face, we contend that team composition disruptions that occur during the completion of a team’s task can be especially challenging. We also argue that it is important to consider different types of team composition changes as they create different demands for team adaptation. Specifically, we assess the effects of loss of a team member and change in team membership resulting from injury substitution. We examine how these two types of team composition disruptions impact coordination and team outcomes (i.e., goals scored) by leveraging data from 2,280 soccer games in the English Premier League. We found that team member loss impaired both team coordination and outcomes while team member substitution only impacted team coordination. Moreover, we build upon and extend existing research that has examined team familiarity by distinguishing between familiarity that is built amongst members on the current team (i.e., current team familiarity) and familiarity that has developed as a result of members working together in prior teams (i.e., prior team familiarity). This distinction appears important as we did not find evidence of a main effect of prior team familiarity on coordination but found evidence of a reversing curvilinear effect of current team familiarity on coordination. Finally, the indirect effect of team member loss on team outcomes through team coordination was more pronounced when teams had low (compared to high) prior team familiarity.

## Introduction

Teams are omnipresent in most industries and carry out much of today’s work. Hence, good team functioning is crucial as it plays an essential role in increasing productivity and corporate success (LePine et al., 2008). As such, the topic of organizational teamwork is a popular one for both popular press and academic research (e.g., Duhigg, 2016). To be clear, academic researchers are primarily interested in understanding the factors that drive team effectiveness with much of this work leveraging the input-process-outcome (IPO) or the more comprehensive input-mediator-outcome (IMO) frameworks (Ilgen et al., 2005). For example, compositional factors (inputs) have been the focus of organizational team research for several decades with this work demonstrating the important role that variables such as demographics, personality, knowledge, skills, and abilities can have on team processes and performance (e.g., Mathieu et al., 2008).

While valuable, much of the work conducted on organizational teams to date has taken a rather static approach in that researchers examine teams at a specific period in time (e.g., Humphrey & Aime, 2014; Summers et al., 2012). The underlying thinking here is that the relationships found at that specific period of time are applicable throughout that team’s entire lifecycle. Unfortunately, such an approach does not adequately take into account the reality that what a team is facing at a certain period of time may not be stable and/or applicable throughout the team’s entire lifecycle as a team may face an event or disruption that may alter its workings and performance. Likewise, events in a team’s past may also impact how a team is able to deal with a disruption currently. An analogy here is that while there are exceptions, too often team researchers have conducted research similar to taking snapshots or still pictures and trying to infer relationships that are better examined by way of data more similar to a video that captures multiple still pictures over time. This inconsistency between research and practice is perhaps even more salient today as teams increasingly work in fast-paced and complex environments in which they regularly face various events that can disrupt their teamwork (Burke et al., 2006; Morgeson et al., 2015). Accordingly, we extend event system theory (EST; Morgeson et al., 2015) as we discuss more fully below by examining multiple types of team composition disruptions as well as how these types of disruptions shape behaviors and other events within a team task episode and therefore, we examine not just how a single disruption will impact performance but delve more deeply into the interchange of disruptive events, behaviors and other events.

As noted by Summers et al. (2012), one of the more common disruptions that teams face is membership change. Unfortunately, the static nature of research mentioned earlier is also relevant to team composition research as voiced by Mathieu et al. (2008) who stated that “teams do not operate in static environments, yet more often than not, team composition research is cross-sectional and provides little insight into temporal dynamics” (p. 442). More recently, Wolfson et al. (2022) noticed that this limitation had not been fully addressed as they stated: “team composition literature has focused on more formal team structures that are stable and bounded as they work toward a shared goal” and that “team composition research has been conducted with either the implicit or explicit assumption that team composition does not change over time” (p. 2).

These quotes are significant as we suggest that while applicable to other parts of the team effectiveness framework, the impact of taking a more dynamic view of teamwork and examining the impact of events is particularly salient within the area of team composition as membership change is a reality for a lot of teams and can have a substantial impact on team functioning as composition changes require adaptation to the new team membership and new role distributions in the team (Arrow & McGrath, 1993; Burke et al., 2006; Shaw et al., 2005). In fact, some have suggested that society is currently experiencing a “Great Resignation” (Cohen, 2021), which is causing many teams within organizations to face increasing composition disruptions as members leave their teams. Similarly, many organizations including those in the technology industry have recently implemented large-scale layoffs (El-Deeb, 2023). As a result, teams are often forced to operate with less individuals than initially envisioned for the team. Alternatively, teams have to adapt to a new member (often drawn from within the organization) joining a team temporarily who may be replacing a team member who has left the organization.

We argue that such team composition changes are therefore a relevant, timely, and impactful disruption that many teams face. Much of the work on team membership changes has examined new members joining a team with no history of working together previously (e.g., Arrow and McGrath, 1993) and this may not be the reality in all situations. As such, within the current study, we aim to address this gap by examining the impact of two distinct types of team membership changes and how such disruptive events impact team coordination and other outcome events. Additionally, given the reality that team members have often worked together within the current team as well as within prior projects, we also examine the impact of team familiarity on the relationship between composition disruptions and team coordination.

Most theoretical and empirical studies that have investigated how teams adapt to team composition disruptions have predominantly focused on disruptions that happen prior to the team’s task execution (e.g., Argote et al., 1995; Lewis et al., 2007; Summers et al., 2012) or between various rounds of task execution (e.g., Kane et al., 2005). Team composition disruptions can just as easily happen during a team’s task execution, and given the focus of research to date, we know relatively little about how teams adapt to team composition disruptions that occur in the midst of task execution during intense and time-limited performance episodes (Ishak & Ballard, 2011; Morgeson et al., 2015). For example, imagine a software development team that has to meet an important project deadline and without prior notice one of the team members leaves the project team shortly before that deadline. We contend that such a composition disruption is different than a composition change that happens in between software development projects.

In this study, we focus on these types of unexpected events that occur during task execution and thus during specific performance episodes by investigating team composition disruptions due to sudden member change. As such, not only do we address a gap in the team composition literature by examining composition disruptions and their impact on team dynamics and team outcome events, but we also examine a unique type of team composition disruption – *within-task composition disruptions*. Thereby, we consider time-limited and specific performance episodes during which the team has to execute their task and simultaneously adapt to disruptive situations (Ishak & Ballard, 2011; Morgeson et al., 2015).

Another extension that we are making with the current study is that we examine different types of within-task composition disruptions in the same study. In so doing, we address calls for research to “flesh out the effects of different types of (disruptions or) triggers” (e.g., Maynard et al., 2015). As such, rather than just examining the impact of membership change as a singular variable, we contend that it is important to delineate the effects that different types of composition changes can have on team processes and outcome events (Bell et al., 2018). We do this by looking at both the loss of a team member as well as the substitution of a team member as a result of injury and how each of these within-task composition disruptions alter team coordination and team outcome events.

It is crucial to better understand how such disruptions affect various team processes so that they can be successfully handled. Here, we focus on team coordination as this is a particularly salient process variable within our sample and has generally been shown to be an important process variable within teams needing to adapt (e.g., DeChurch & Haas, 2008). In fact, coordination is one of the most important behaviors for teams adapting to changing circumstances, and enables a team to function flawlessly as a unit and perform well (e.g., Grote et al., 2010; Manser et al., 2008). Coordination generally refers to teams organizing their resources, activities and responses in order to integrate, synchronize, and complete their tasks (Cannon-Bowers et al., 1995). In our study, we focus on coordination as an active process during a specific performance episode, and thus define it as synchronizing the timing and sequence of interdependent actions (Brannick et al., 1993; Marks et al., 2001). We envision that coordination will be impacted by the two different forms of team composition disruptions examined and will be a salient mediator of the relationship between such composition disruptions and team outcome events.

Given our emphasis in the current study on different types of team composition disruptions, we suggest that it is also important to consider other team composition factors in parallel to our examination of the influence of composition disruptions. In particular, there is evidence to suggest that team familiarity (i.e., team members' shared experience working together; Espinosa et al., 2007; Reagans et al., 2005) contributes to team coordination and performance (e.g., Okhuysen, 2001; Taylor & Greve, 2006) and we suggest that team familiarity could be salient for teams facing team composition disruptions. Accordingly, we consider team familiarity to be an important contextual determinant of successful adaptation to team composition disruptions. While prior research has shown that familiarity facilitates team performance through its impact on team coordination (e.g., Reagans et al., 2005), we know very little about team familiarity’s role in shaping the relationship between team composition disruptions and coordination. Likewise, we build upon prior considerations of team familiarity to not only examine the level of familiarity that has been gained through involvement in the current team but also the level of familiarity that team members have gained in prior teams that members may have worked on together.

As such, within the current study, we make two primary contributions. First, drawing primarily upon EST (Morgeson et al., 2015), we investigate the effects of two different forms of team composition disruptions (i.e., loss of a team member and substitution of a team member resulting from injury) on coordination and team outcome events. We expect both forms of composition disruptions (i.e., an unexpected membership reduction such as loss or a membership change that results in different membership in response to events such as injury substitution), to be harmful for coordination and outcome events because they either reduce or alter the resources possessed by the team. However, we envision that a loss of a member will have a more deleterious effect on team dynamics and team outcome events. By distinguishing between these two forms of team composition disruptions, we simultaneously seek to shed light on how they might differ in their severity and thus impose different challenges to the teams’ adaptation. As such, our study extends prior work on team disruptions which has only examined single types of disruptions (e.g., Lewis et al., 2007; Summers et al., 2012). We thereby address the call by Maynard et al. (2015) to consider “the nature of the triggers that give rise to team adaptation and how such triggers may shape the specific processes that need to be adapted by the team” (p. 13). Likewise, rather than simply looking at how such disruptions shape team outcomes, we extend empirical examinations of EST by looking at how these two distinct forms of composition disruptions shape team behaviors and other events within a team performance episode. As such, we delve deeper into the impacts of disruptive events on team functioning than has been done by most examinations of EST.

Second, our study addresses calls for research to include considerations of temporal factors within studies of organizational teams (e.g., Kozlowski, 2015; Leenders et al., 2016) in two ways. For starters, we examine the effect that team composition changes have on team coordination and outcome events (i.e., goals scored) within a single team performance episode (i.e., professional soccer games) and do so across three seasons of the English Premier League (EPL). Additionally, we include two distinct forms of team familiarity which are built over differing time horizons. Specifically, we examine familiarity that is gained from members being on the current team together (i.e., current team familiarity) as well as shared experiences team members have had on prior teams (i.e., prior team familiarity). We contend that this is a needed extension of team familiarity research as there are numerous instances where these two distinct forms of familiarity may be relevant. For instance, think of a motion picture production team which works in various locations over several months capturing what is needed for their current project, then disbands. In later projects, certain team members may have experience working with their fellow team members on prior movie projects. Many studies within the familiarity research literature have only examined shared experiences that members have working together on the current team or project (e.g., Sieweke & Zhao, 2015) and have shown the salience of that type of team familiarity. However, conceptualizing familiarity as only emerging from the current team ignores the fact that members may have worked together on prior projects as well and therefore come to the current team with some level of familiarity already established. Research conducted to date has not examined whether this more distal form of team familiarity is more/less salient in shaping team dynamics and performance – an important gap that we will address with the current study.

### Event System Theory Grounding and Contribution

As noted previously and articulated by Morgeson et al. (2015) too often research focuses on stable and enduring *features* present within organizations and examining how these features shape various dependent variables. While valuable, these authors advocate for more attention on *events* as events can impact both behavior and features and thereby affect organizations across time. Accordingly, Morgeson et al. (2015) put forward their seminal work on EST where they referenced the work of Floyd Allport and others in suggesting that events are “discrete, discontinuous “happenings,” which diverge from the stable or routine features of the organizational environment” (pg. 519) and require attention as such events play “a major role in shaping thoughts, feelings, and actions” (pg. 515). While the EST is gaining in popularity, much of the work empirically examining this theory to date has focused on individual level phenomenon. Here, we examine the impact of events on team level relationships. Likewise, much of the work conducted to date has examined the impact that a singular event has on a single behavior or some measure of performance (e.g., McFarland et al., 2020). Such an approach is valuable as a first step in examining EST, however, it is not in keeping with the complete underlying logic presented by Morgeson et al. (2015).

Namely, in their theorizing they suggested that events are influential within organizations as they can impact (1) behaviors, (2) features, and/or (3) other events. As such, within the current study, we examine two distinct team composition disruptions and how such disruptions affect team behaviors (i.e., coordination) as well as looking at how such disruptive events play within a broader consideration of other events (i.e., goals scored) within an overall team task episode (i.e., a soccer match). Accordingly, we argue that we extend the work that has been conducted to date regarding EST, as we examine events in a more comprehensive way than previously done. As such, we argue that we are following the recommendations of Mathieu et al. (2008) as we embrace “… the complexity of current team arrangements” (page 463) as many teams do not encounter a single event but instead encounter various events within a single task episode and this is the approach we take within the current study.

Likewise, within our theorizing presented here, we rely heavily on Morgeson and colleagues’ (2015) work which lays out various factors that shape an event’s strength. Namely, they suggest that the event characteristics of novelty, disruption, and criticality shapes whether an event rises to the level where action is needed and they argue that as an event’s strength is increased based on these three characteristics, the more likely it will require the team to change or create behaviors, features, and events. *Novelty* “reflects the extent to which an event is different or varies from current and past behaviors, features, and events, thus representing a new or unexpected phenomenon” (Morgeson et al., 2015, pg. 520). Likewise, *disruption* “reflects a discontinuity in the environment” (pg. 521) and as a result, “things do not continue the way they did prior to the event” (pg. 521). Finally, *criticality* reflects “the degree to which an event is important, essential, or a priority” (Morgeson & DeRue, 2006: 273).

### Team Composition Disruptions as Events

Some events that teams are confronted with can be anticipated (e.g., launch of a new technological tool), while other events arise unexpectedly (e.g., sudden illness of a team member). Events are especially disruptive when they require immediate action for which the team cannot prepare (Burke et al., 2006; Morgeson & DeRue, 2006). Disruptive events require a team to adapt its behavior to achieve its goals and avoid breakdowns in team functioning and performance, which may entail more stability and/or flexibility in team processes (Grote et al., 2018; Louis, 1980; Maynard et al., 2015; Morgeson et al., 2015).

While team membership changes have been examined in the literature, many of these studies have manipulated these compositional events by altering membership prior to a task challenge or in between a series of task challenges (e.g., Argote et al., 1995; Arrow & McGrath, 1993). Granted that these examinations are interesting and valuable to consider, however, such work is distinct from membership changes that happen during a team’s task completion (i.e., within-task composition disruptions) which are more likely influential on the team’s behaviors and features. Previous team research has shown that time bound events such as disruptions can take on multiple forms and impact team functioning in different ways (Christian et al., 2017; Morgeson & DeRue, 2006; Morgeson et al., 2015). They can affect a team’s taskwork and/or teamwork and have been categorized into task-based and team-based disruptions accordingly (Maynard et al., 2015).

Task-based disruptions arise from the environment and confront the team with unexpected complications regarding the task or with nonroutine events that require the team to adapt (Christian et al., 2017; Marks et al., 2001). Team-based disruptions arise from within the team either prior to a team’s task execution (e.g., due to turnover) or during a team’s task execution (e.g., due to unexpected member dismissal or substitution). Here, we focused on a particular type of team-based disruption, specifically team composition disruptions that occur during the completion of a given task and how such events alter team outcome events (i.e., goals scored) through their impact on team behaviors (i.e., coordination).

### Team Composition Disruptions and Team Outcome Events

Extant research investigating how changes in team composition affect team performance has mostly examined turnover as a form of membership change that takes place prior to the team’s task execution (e.g., Argote et al., 1995; Lewis et al., 2007). For instance, Van der Vegt et al. (2010) found that turnover had a negative impact on team performance due to effects on key interaction processes (e.g., team learning), while DeRue et al. (2008) examined how different downsizing approaches affected team adaptation and performance and found that eliminating hierarchy from the team led to better team adaptation and, in turn, performance. Research has generally acknowledged that membership change can have positive and negative effects on teamwork (Arrow & McGrath, 1993, 1995; Lewis et al., 2007), but studies have more often considered member changes in teams as harmful disruptions (i.e., Arrow et al., 2000; Summers et al., 2012).

In this study, we focus on team composition disruptions that occur during task performance as an even more critical and challenging situation for teams (e.g., Morgeson & DeRue, 2006). We examine the effects of unplanned changes in team composition on team coordination and team outcome events in time-limited performance episodes where fast adaptation is of utmost importance. Additionally, we distinguish two forms of team composition disruptions within the same team context to examine whether their effects vary due to differences in their severity (Christian et al., 2017; Maynard et al., 2015). We juxtapose disruptions caused by the *loss* of a team member with disruptions caused by the necessity to withdraw a team member as a result of injury and *substitute* that individual with another team member. Both forms of disruption (i.e., loss and substitution) take place during an ongoing performance episode (i.e., a soccer game) and thus have an immediate impact on teamwork and can lead to a breakdown in team performance. This is relevant as Hausknecht & Holwerda (2013) suggest that any gains from composition changes may not accrue until later in the team’s lifecycle and that detrimental effects of such changes may be more pronounced in the near term. More recently, Li and van Knippenberg (2021) also suggest that membership change “initially disrupts team cognitive, behavior, and interpersonal processes and states, and as a result negative performance effects may initially dominate” (pg. 578). Given that we are examining the effect of within-task composition disruptions, and the negative effects that appear to happen initially following a membership change, we anticipate that our within-task composition changes will more likely lead to such negative consequences.

As discussed previously, our study also extends empirical examinations of EST because we examinate the impact of two distinct within-task composition disruptive events and the role that they have on team outcome events (i.e. goals scored) which is in keeping with Morgeson and colleagues’ (2015) suggestion that events impact other events even though much of the work on EST has not examined this complex reality faced by many teams. Like Trainer et al. (2020) suggest, we leverage EST to examine the within-task composition disruptions studied here. Namely, the loss of a member as well as needing to unexpectedly substitute a member are disruptive to the team’s current state and requires alterations within the team which may result in the team needing to engage in “effortful information processing and changes to existing behaviors and features or the creation of new behaviors, features, and events” (Morgeson et al., 2015: 521). Likewise, relying on Morgeson and colleagues’ (2015) theorizing around event criticality, while all composition changes may be critical, we argue that a membership change experienced within-task is likely even more critical as it is likely to have an influence on events in the future (e.g., Hoffman & Ocasio, 2001; Pirola-Merlo et al., 2002). Likewise, changing membership in the midst of completing a task is apt to have a greater impact on the attainment of team goals (e.g., Ballinger & Rockmann, 2010; Weick, 1988).

In part, composition changes are disruptive as they often lead to the need of changes in patterns of interrelationships among members (e.g., Wolfson & Mathieu, 2017) as someone may need to take on new roles to replace the loss of a member. Expending effort in this way may distract the team from what was working prior to these within-task composition disruptions and thereby result in decreases in team performance. Likewise, as argued by Wolfson et al. (2022), emergent states such as shared cognition can be negatively impacted with membership changes and there is a wealth of literature evidencing the important role that emergent states play in shaping team performance (e.g., Mathieu et al., 2008). Similarly, while not the focus of their work, Reeves (2008) provided evidence within a healthcare setting that changes in group membership undermined cohesion and the team’s ability to collaborate effectively which impeded the team’s progress. Furthermore, given that both of the within-task composition disruptive events examined here are unexpected, their effects are more likely to be negative and pronounced as compared to dynamic composition events that are more likely anticipated (e.g., Wolfson et al., 2022). Likewise, both forms of team composition disruptions examined here can be considered to require team members to invest additional resources either by taking over additional work or reallocating roles and workload to keep up their performance. As a result of the cumulative effects that within-task composition disruptions may have, we argue that they likely lead to distractions from the main task, resulting in lower performance.

Hypothesis 1a Team composition disruptions characterized by losing a team member are negatively related to team outcome events.

Hypothesis 1b Team composition disruptions characterized by substitution of a team member due to injury or illness are negatively related to team outcome events.

### Team Composition Disruptions and Team Coordination

Extant research has investigated changes in team structures and composition by focusing on aspects such as varying demographic diversity (Harrison et al., 2002), personality mixes (Peeters et al., 2006) or the role of strategic core team members (Summers et al., 2012). Most of these factors have been found to be related to team outcomes. Only a few studies have investigated intermediary processes of how teams adapt their behavior to member change (Lewis et al., 2007; Pisano et al., 2001). This is unfortunate and not in keeping with the theorizing of Morgeson et al. (2015) who strongly suggest that events are apt to shape team behaviors especially as event strength rises. In fact, these authors cite Rescher (1996) to emphasize the importance of studying the impact of events on processes which we do here with our examination of team coordination. Namely, they quote Rescher (1996) as stating that “process has primacy over things. Substance is subordinate to process: Things are simply constellations of processes” (p. 2). Accordingly, in an attempt to extend current work addressing EST, we investigate team coordination as a mediator of the relationship between both forms of team composition disruption that arise during a team’s task execution and team outcome events. More precisely, we focus on team coordination because of its central role in most team effectiveness frameworks (e.g., Marks et al., 2001) and the fact that it has been argued to be central in considerations of team membership change (e.g., Summers et al., 2012). Coordination has been defined as “the process of orchestrating the sequence and timing of interdependent actions” (Marks et al., 2001: 367–368) and within our study, team coordination is operationalized as completed passes between teammates as this is one of the most essential activities for soccer teams to perform as a unit in order to be successful (e.g., Garganta et al., 1997). Good passes need coordination and anticipation between team members, while unsuccessful passes can be seen as failed intended coordination and has been operationalized similarly in prior studies (Sieweke & Zhao, 2015).

The loss or substitution of a team member may lead to a situation in which team members are no longer able to rely on the functioning of their previous workflow (e.g., accurate passing between players). For example, the validity of previously established transactive memory systems or shared mental models might diminish and require time to be reestablished in the new team composition (Lim & Klein, 2006; Mathieu et al., 2000). As explained above, when team composition disruptions arise, teams must actively redistribute roles and responsibilities to build up a shared understanding of each other’s behavior and maintain effective team coordination (Christian et al., 2014; Marks et al., 2000; Rico et al., 2008). Because redistributing roles and responsibilities needs time and requires the team to invest additional resources (Ogungbamila et al., 2010; Steiner, 1972), changes in team composition resulting from either a loss or substitution of a team member may lead to process losses, which result in a lower level of team coordination.

Hypothesis 2a Team composition disruptions characterized by losing a team member are negatively related to team coordination.

Hypothesis 2b Team composition disruptions characterized by substitution of a team member due to injury or illness are negatively related to team coordination.

While the two hypotheses above suggest that both team composition disruptions will have negative implications for team coordination and team outcome events, neither hypothesis speaks to the relative effect the respective disruptions may have. Accordingly, in hopes of addressing Christian et al. (2017) as well as Maynard and colleagues’ (2015) call for a more thorough investigation of the nature of disruptions faced by teams, we consider the relative effect of the two types of team composition disruptions. As detailed by Morgeson and colleagues (2006; 2015), events can be evaluated based on their urgency, duration, and criticality. Event urgency “reflects the degree to which the team must respond immediately to an event in order to either capitalize on its occurrence or mitigate its negative consequences” (Morgeson & DeRue, 2006, p. 273). The two types of team composition disruptions examined here (i.e., loss and injury substitution) both have to be dealt with immediately within the context of EPL soccer games. The next dimension outlined within EST is event duration, or how long an event lasts. Given that we investigate the team’s ability to coordinate and perform following both types of composition disruptions, in some respects, we examine the duration over which the consequences of these events are salient for the team within our measure of both loss and substitution as we capture when the two within-task composition disruptive events (i.e., loss and substitution) occur within the game (i.e., early or later in the game). As such, by way of our measure, the longer that a team has to deal with either a loss or injury substitution, the more negatively team coordination and team outcome events will be impacted. That said, the final dimension detailed within EST, criticality is likely the most relevant to our theorizing here. As discussed by Morgeson and DeRue (2006, p. 273), criticality “reflects the degree to which an event is important, essential, or a priority to the team.” A loss of a team member results in the team being down a teammate and therefore possessing less resources, skills, and abilities than prior to the loss of the member. This is likely a more severe consequence within the team as compared to situations where teams must adapt to a replacement teammate entering a game as a substitute for an injured player. While the substitution will undoubtedly be challenging to the team as they will have to reallocate roles and get used to a new member and their skills and abilities relative to the individual that they replaced, it will not be as critical a situation as having to play with only 10 players versus 11 as is the case with the disruption caused by team member loss. Accordingly, we contend that:

Hypothesis 3a A loss of a team member will have a more severe negative effect on team outcome events than an injury or illness substitution.

Hypothesis 3b A loss of a team member will have a more severe negative effect on team coordination than an injury or illness substitution.

### Team-Coordination and Team Outcome Events

As described previously, coordination generally refers to teams organizing their resources, activities and responses in order to integrate, synchronize, and complete their tasks (Cannon-Bowers et al., 1995). In our study, we focus on coordination as an active process during a specific performance episode, and thus define it as synchronizing the timing and sequence of interdependent actions (Brannick et al., 1993; Marks et al., 2001). Beyond being a particularly salient construct within our sample, coordination is a central component in almost all team effectiveness frameworks (e.g., Mathieu et al., 2008). Likewise, coordination is one of the most important factors for a team to adapt to changing circumstances and to function flawlessly as a unit (Burke et al., 2006; Grote et al., 2010; Manser et al., 2008; Rico et al., 2008).

This relationship is especially true for teams performing highly interdependent tasks (Fiore et al., 2001; Okhuysen & Bechky, 2009). Namely, research has documented the important role of team coordination in shaping performance results in contexts as diverse as healthcare (e.g., Henrickson Parker et al., 2018) and construction road crews (Tesluk & Mathieu, 1999). Moreover, studies have shown that effective team functioning is integrally linked with team productivity and success (Antoni & Hertel, 2009; LePine et al., 2008). Following extant team literature, we propose that team coordination is positively related to team outcome events.

Hypothesis 4 Team coordination is positively related to team outcome events.

### Team Familiarity and Team Coordination

In this study, we investigate *current team* and *prior team* familiarity in combination with team composition disruption events and team coordination. We suggest that team familiarity is an important contextual feature that requires more attention when looking at team coordination. Team familiarity is defined as the shared experience a team has working together (Espinosa et al., 2007; Reagans et al., 2005). Typically, team performance improves during the time a team works together (e.g., Huckman et al., 2009). This has been shown in various settings such as in software teams (Faraj & Sproull, 2000) and in experimental teamwork settings (e.g., Gruenfeld et al., 1996). Even short periods of shared work history have been shown to improve team performance in flight simulation teams (Kanki & Foushee, 1989). When team members go through various situations together, they learn how other members react to different situational demands and what capabilities and action-tendencies each member has. Thus, team members learn to combine information about a current situation with previously acquired taskwork and teamwork knowledge and learn to foresee actions of team members (Eccles & Tenenbaum, 2007). Over time, teams establish shared knowledge which helps them improve their communication (Krauss & Fussell, 1990) and augment their shared understanding of taskwork and teamwork (Moreland and Myaskovsky, 2000).

Increasing team familiarity does not only result in a more profound shared team-knowledge but it also enables complex coordination patterns that a team with little or no shared experience lacks (Cramton, 2001; Dalal et al., 2017; Luciano et al., 2018; O'Neill & Rothbard, 2017). Due to these mechanisms, team familiarity is seen to be closely related to interaction processes between team members that allow teams to improve their coordination over time (Kraut et al., 2005). Moreover, team members are able to anticipate others’ actions and adjust their own behavior to these anticipated actions, enhancing coordination (Okhuysen & Bechky, 2009; Rico et al., 2008). By means of coordination, teams can develop routines and take correct actions without consciously considering alternatives (e.g., Hollingshead, 1998; Lewis, 2003). Therefore, team familiarity is seen to have positive effects on team coordination, which has been also shown empirically (e.g., Espinosa et al., 2007; Reagans et al., 2005).

In part, the positive effects of familiarity are attributable to social identity theory (e.g., Tajfel & Turner, 1986) which suggests that individuals build up identity to groups they belong. Stated differently, identity can be viewed as one’s sense of belonging to a social group. This sense of belonging can be tied to the relationships that individuals have with their teammates. As such, to the extent that individual members have worked together previously and thereby are more familiar with one another, social identity should be enhanced. This is pertinent as enhanced social identity has been tied to enhanced levels of knowledge sharing and performance within teams (e.g., Kane et al., 2005).

However, considering recent studies, there is reason to believe that the effect of team familiarity might evolve differently over time. Berman et al. (2002), for example, found that the positive relationship between shared experience and team performance in basketball teams diminished over time. The authors argue that having established tacit knowledge can lead to a state in which team members assume that they have the ability to coordinate implicitly with the other team members. However, this feeling of familiarity and the established shared knowledge within a team can also have downsides in which teams start relying too much on routines and can be surprised by sudden deviations. The reasoning of Berman et al. (2002) aligns with the study of Sieweke and Zhao (2015) who found a U-shaped effect of team familiarity on team coordination errors in basketball teams. The authors as well as other authors that study familiarity on a broader level (i.e., Zheng & Yang, 2015) argue that team familiarity decreases coordination errors to a certain degree but too much familiarity may encourage teams to develop habitual routines. These routines might result in heedless implicit coordination or rigidity of well-rehearsed behavior that can result in coordination breakdowns.

Building on the study of Sieweke and Zhao (2015), we propose that team members initially build up shared knowledge by getting more familiar with one another and thereby achieve synchronicity that leads to good team coordination. However, once a profound shared team knowledge has been established, it becomes more difficult for team members to amend the internalized knowledge that they have about the interdependent behaviors of the other team members. Thus, we follow Sieweke and Zhao (2015) and hypothesize that, after a certain point, team familiarity will have detrimental effects causing a decrease in coordination.

Hypothesis 5 There is an inverted U-shaped relationship between team familiarity and team coordination. As team familiarity increases, team coordination will also improve but to a lesser extent. This relationship will continue up to a tipping point, after which a further increase in team familiarity will decrease team coordination.

### The Moderating Effect of Team Familiarity

In addition to its main effect on coordination, we expect team familiarity to be a salient contextual feature when it comes to helping teams adapt their coordination to team composition disruptions. Team composition disruptions often occur unplanned. In the case of losing a team member or substituting a team member as a result of an injury or illness, teams usually need to quickly adapt to the changes in team size or composition by redistributing roles and adapting strategies to regain stability and remain coordinated within the same performance episode (Grote et al., 2018; Ogungbamila et al., 2010). In these situations, adaptation is especially crucial because a team is in the midst of a performance episode with little opportunity for corrections (Ishak & Ballard, 2011). Thus, there is little to no time for the team members to elaborate and reflect about the new situation (LePine, 2005; Schmutz et al., 2018). Extant research has shown that familiarity can help teams to effectively coordinate (e.g., Espinosa et al., 2007; Reagans et al., 2005; Sieweke & Zhao, 2015). To our knowledge, our study is the first to also investigate team familiarity as a contextual variable that can help teams adapt to unforeseen team composition disruptions.

We expect that team members who have acquired substantial common team knowledge in the past by sharing a variety of experiences and gaining familiarity with one another, are better able to adapt their coordination to team composition disruptions during time-limited performance episodes than team members who are not (or less) familiar with each other. While there is limited research that has examined team familiarity as a contextual feature that can moderate relationships, we rely on prior work that has evidenced that shared experiences between individuals can build up team human capital (e.g., Chillemi & Gui, 1997) which can improve team performance especially when faced with challenging events. Likewise, Weick and Roberts (1993) suggest that familiarity leads to “heedful interrelating” which sets a baseline for team action. Related, team experience and familiarity has been shown to assist with the development of transactive memory systems which has been tied to enhanced team performance (e.g., Wegner, 1987).

Therefore, as familiarity increases within a team, it should provide a buffer against the challenges that emerge as a team faces within-task composition disruptions. More precisely, if a team unexpectedly loses a member during task completion, this puts pressure on the team and may otherwise hinder the team’s ability to coordinate their actions. However, in the face of such an event, if the remaining team members have a vast amount of experience working together, their inherent familiarity should enable them to rely on their transactive memory systems and human capital that has been built up over time to reduce the negative consequences of this member loss. In such situations, team members will know who they can rely upon to take up slack for the missing team member and will better know who should serve which roles in the absence of a team member.

Similarly, when a team has to unexpectedly substitute one team member for a replacement, all things being equal this can also impair the coordination of the team in the near term as they have to learn to adjust to the new member (e.g., Li & van Knippenberg, 2021). Again, if such a replacement occurs within a team that has a great deal of familiarity with one another, the team has the shared cognition and human capital that should minimize the negative effects of such a substitution. Likewise, enhanced levels of familiarity have been connected to higher levels of trust among team members (e.g., Granovetter, 2018; McEvily et al., 2003) which should serve the team well when faced with either type of within-task composition disruption and this trust built upon enhanced familiarity can help to diminish the negative effects of team composition changes. Our line of thinking here is in keeping with the proposition recently introduced by Trainer et al. (2020) when they argued that “factors that allow teams to capitalize on prior experiences after a team membership change event can decrease the disruptiveness of the event” (pg. 241). We envision team familiarity doing just that – reducing the disruptiveness of each of the within-task composition disruptions examined here and thereby the negative effects of these membership changes on coordination being minimized as familiarity increases. Based on these arguments, we contend that the negative effects of both types of within-task composition disruptions (i.e., loss and substitution) on team coordination will be mitigated as team familiarity increases.

Hypothesis 6a Team familiarity moderates the relationship between team composition disruptions characterized by losing a team member and team coordination, such that as team familiarity increases, the relationship between the team composition disruptions and coordination will be attenuated.

Hypothesis 6b Team familiarity moderates the relationship between team composition disruptions characterized by substitution of a team member as a result of an injury or illness and team coordination, such that as team familiarity increases, the relationship between the team composition disruptions and coordination will be attenuated.

By integrating the hypotheses above, we hypothesize that both types of within-task composition disruptions negatively affect team outcome events in the near term as a result of the disruption such events have on cognitive, behavioral, and interpersonal processes (e.g., Li & van Knippenberg, 2021). In the current study, we suggest that coordination will be a primary means by which the negative effects of both types of within-task composition disruptions likely impair team outcome events. In particular, the workflows and coordination practices that were in place prior to the composition changes will likely have to be altered with one member less (i.e., loss) or with different team members (i.e., substitution) and reestablishing effective coordination practices will take time to establish and therefore, coordination processes will likely be negatively impacted by both types of within-task composition disruptions examined here. Accordingly, within the current study, we are suggesting an indirect effect of team composition disruptions on team outcome events (i.e., goals scored) via the effect such disruptions have on team coordination.

**Figure 1.**
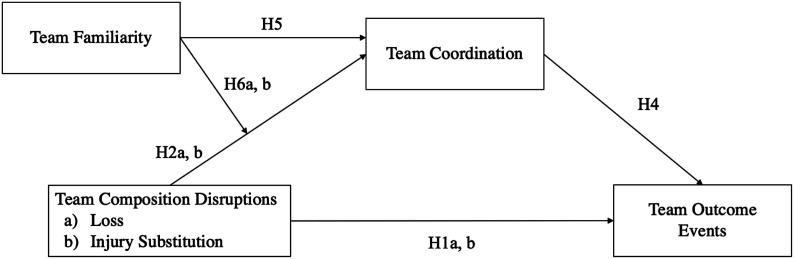
Study model. *Note.* While not depicted in the figure, hypothesis 3 suggests that there is a difference in the severity of the two types of team composition disruptions (loss vs. injury substitution) on team coordination and outcome events. Hypothesis 7a and 7b describe the moderated mediation of team composition disruptions on team outcome events through team coordination, which is moderated by team familiarity.

Simultaneously, we introduced team familiarity as a contextual variable that can facilitate team adaptation and thus help maintain team coordination when team composition disruptions arise and thereby, we suggest that familiarity will help to reduce the negative effects that the composition changes have on team coordination and ultimately impacting team outcome events. Accordingly, we are hypothesizing a conditional indirect effect by arguing that team familiarity moderates the indirect effect of team composition disruptions on team outcome events via team coordination. As such, we introduce a moderated mediation model to assess how and under what conditions of team familiarity, a given effect exists between team composition disruptions, team outcome events and team coordination (e.g., Peñarroja et al., 2015). In large part, we contend that familiarity will allow the team to preserve adequate transactive memory systems, human capital, and trust levels that will help the team to adjust as needed in the face of such unexpected composition events. That said, it may not be possible for the team to completely overcome the process losses incurred by a team facing the team composition disruptions examined here even though team familiarity should be helpful. Therefore, we propose that team familiarity will moderate the indirect effects of team composition disruptions caused by loss or substitution of a team member on team outcome events through team coordination (see Figure 1).

Hypothesis 7a The indirect effect of team composition disruptions characterized by losing a team member on team outcome events through team coordination is moderated by team familiarity. The indirect effect is dampened as team familiarity increases.

Hypothesis 7b The indirect effect of team composition disruptions characterized by substitution of a team member as a result of an injury or illness on team outcome events through team coordination is moderated by team familiarity. The indirect effect is dampened as team familiarity increases.

### Time Horizons and Conceptualization of Team Familiarity

Research so far has measured team familiarity in a variety of ways without considering differentiated time horizons over which familiarity was obtained within the team and as a result, research on familiarity has not fully acknowledged the variety of experiences that could potentially be shared over different contexts. Most prior research considering team familiarity has only examined the familiarity derived from working together on the current team. For instance, the work of Sieweke and Zhao (2015) which looked at the effect of familiarity on team coordination errors within professional basketball teams measured team familiarity as the number of years each pair of players on the team played together on the current team. As such, this prior study as well as most other studies that have examined team familiarity have not considered the fact that team members may have also gained familiarity while working together on a prior different team. This reality was raised by Mathieu et al. (2014, p. 150) when they stated that “At issue then is that research to date has not adequately accounted for the fact that, for each team configuration, there is a network of intermember histories of working together.” We argue that it is important to not just consider the histories of working together on the current team but to consider all instances of working together in the past even when they occur on other teams.

In order to distinguish these different forms of team familiarity, in this study, we refer to experience working together on the current team as *current team familiarity*. However, we suggest that it might be equally important to consider the time team members have spent together in previous teams as there is reason to expect that the variety of experience and shared knowledge gained through such experiences on other teams is carried over when members work together in subsequent teams (e.g., Goodman & Shah, 1992; Mathieu et al., 2014). Thus, it is essential to recognize that individuals may also build familiarity with one another in prior teams and organizations - which we refer to as *prior team familiarity*.

As previous research has not considered the distinction between current and prior team familiarity, we did not formulate specific hypotheses about differential effects, but rather a research question.

Research Question 1 Do the effects of team familiarity on coping with team composition disruptions differ depending on whether it is current team familiarity or prior team familiarity?

## Methods

### Sample and Procedure

Given the relevance to our research questions and the value of leveraging sports teams for the study of groups and teams in organizations (e.g., Quigley et al., 2022), in order to examine our hypotheses, we relied on an existing database of soccer teams within the EPL and combined this data with a dataset on players’ career trajectories established and used by Velema (2016). The former dataset was obtained from *Opta*, a leading sports data company that provides detailed data on various sports. The data is collected in specialized data collection centers and contains information on general statistics of the game (e.g., accurate passes, total passes, goals), as well as information on disruptive situations (e.g., suspensions and injuries). The latter dataset was initially used for a study about player migratory trajectories in the global soccer labor market (Velema, 2016, 2018). This dataset contains information on players’ career trajectories including transfers from one team to another and was obtained from *Transfermarkt* a German website that serves as a reliable data source for academic investigations in the field of professional soccer (Bryson et al., 2012; Herm et al., 2014).

In total, our sample consisted of three EPL seasons (i.e., 2006/2007; 2007/2008; and 2008/2009). The EPL consists of 20 teams that play 38 games each per season. Within each season, each team plays twice against every other team in the league, which results in 380 total games per season and a total number of 1,140 observed games for three seasons. That said, our unit of analysis is each team that participates within each of these games. This means that in the total sample, each game results in two outcomes – one for each team because all variables are assessed on a team per game level (i.e., goals per team in each game, team coordination per team in each game). Therefore, the total sample size is *N =* 2,280 observations. We account for the cross-classified data structure in our analytical approach (see paragraph Data structure and analytical approach). Due to promotion and relegation, the 20 teams playing in the EPL each season change. At the end of each season the worst teams transfer out of the EPL into a lower division and some teams from the lower division enter the EPL. Because we included data from three seasons the dataset contains data from 26 different teams over the course of the three seasons.

### Measures

#### Team Composition Disruptions

We assessed two forms of team composition disruptions. First, when a team member leaves the field during the game due to a yellow-red or red card (suspension), which we considered as team member loss. In this case the team size decreases from 11 to 10 players and thus the workforce is smaller (*n*-1). Second, when a team member forcefully leaves the field due to an injury during the game but gets substituted by another player, which we considered as team member substitution. In this case, the team size remains the same (i.e., 11) but there is a forced and unplanned change in the team membership. While there are expected substitutions during games made by the coach for strategic reasons, we did not examine these substitutions as we are interested in unexpected disruptions in team composition and thus, we focus on substitutions required by player injury as these substitutions are unanticipated by the team and therefore more disruptive.

To assess the duration of the disruptive situation, we measured the number of minutes the team had to play under the distinct team composition disruptive situations. Therefore, we subtracted the minute in which the respective disruption (i.e., loss or substitution) occurred from the total game time to receive our variables: *Loss Disruption Duration* and *Injury Substitution Disruption Duration*. Accordingly, a team that encounters one of these disruptions earlier in the game has a longer duration while a team composition change that happens toward the end of the game will have a lower duration value.

#### Coordination

Coordination was measured by objectively observed activities essential for soccer teams to perform well. Past studies have shown that passes are one of the most important features among soccer and other sports teams (Garganta et al., 1997; Sieweke & Zhao, 2015). Good passes need coordination and anticipation between a minimum of two players, while unsuccessful passes can be seen as failed intended coordination (Sieweke & Zhao, 2015). For a team to be able to pass among members, they need to be in offensive play. Hence, we considered coordination as the proportion of the number of accurate passes to the number of total passes.

#### Team Outcome Events

Following Grund (2012) who used similar data to ours, we used the number of goals the team scored in the given game as a measure for offensive team outcome events. According to Grund (2012, p. 684), “limiting the analysis to offensive performance seems reasonable because the data only allows for the investigation of the orchestration of offensive team play (i.e., when a team possess the ball and players pass).”

#### Team Familiarity

To get a team familiarity measure, we followed the approach by Sieweke and Zhao (2015) and adapted their measure to the setting of our study. First, we calculated the number of months a player played with each of the other team members. Second, we multiplied this value by the number of minutes a player played in the particular game. This allowed us to consider each player’s importance for the team, because the players vary in their amount of playing time. Third, we summed up these scores for all members of a team to achieve a team score. Finally, we divided this team score by the number of players minus one to get our final team familiarity score. For scaling reasons, we divided this value by 100. A detailed breakdown of our familiarity measure can be found in the Supplemental Material.

Further, we distinguished between *prior team familiarity* and *current team familiarity*. While prior team familiarity is a cumulative measure and considers the familiarity over the complete time the players played with each other in any team since their entrance to their professional soccer career up to the current season under investigation, current team familiarity only considers the time players have played together within the current season that is under investigation. Thus, prior team and current team familiarity differ regarding the time span over which familiarity has been developed between team members. More importantly, the two measures also differ regarding the experiences that team members made with each other in different team contexts and settings.

#### Control Variables

It is well-established that there are advantages in being the home team (i.e., Carmichael et al., 2000). We therefore controlled for the place of the game by considering whether it was a home game for the team under investigation or a game that was played in the opponent’s stadium (Away game = 0; Home game = 1). Furthermore, we controlled for the strength of the team as well as their opponent in every game by considering the team’s standing (i.e., ranking) within the league at the beginning of the given game (i.e., González-Rodenas et al., 2019). Therefore, we took the number of points of the given team before the game and divided it by the maximum possible points that could be achieved up to the given game (team’s standing). Analogously, for the opponent’s team we took the number of points of the opponent before the given game and divided it by the maximum possible points up to the given game (opponent’s standing). Finally, all results were analyzed with and without controlling for disruptions (i.e., loss and substitution of a team member) that may have taken place in the game within the opponent’s team. However, these additional control variables did not change the results in any notable way. Finally, to rule out systematic temporal effects, we included the season (dummy variables) and the game number during the current season as additional control variables.

#### Data Structure and Analytical Approach

Data from the EPL is hierarchical in nature. However, the outcome variables (i.e., goals and coordination per team per game) are nested in more than one higher-level cluster simultaneously and therefore cross-classified (Claus et al., 2020). We see the outcome variables (i.e., goals and coordination) as events that are nested in games and also in teams. Each game results in event outcome measures for the home team and the away team (goals, coordination per team per game). Therefore, we treat our level 1 event outcomes as nested in games (level 2a) and teams (level 2b). To address this, we performed hierarchical linear modeling controlling for both higher-order categories (i.e., games, teams) using the packages “lmerTest” (Kuznetsova et al., 2022), “multilevel” (Bliese et al., 2022) and “nlme” (Pinheiro et al., 2022) in R (R Core Team, 2020). The intra-class correlation coefficient (ICC 1) for level 1a (games) for outcome events was .004 and .17 for coordination, meaning that games explain .4% of the variance in outcome events and 17% for coordination. The ICC 1 for level 2a (team) for outcome events was .05 and .37 for coordination, meaning that teams explain 5% of outcome events and 37% of coordination. To analyze the full model for the moderated mediation effects predicted in Hypotheses 7a and 7b, we computed bootstrapped confidence intervals with 10,000 bootstrap samples (Preacher & Hayes, 2004) by using the SPSS macro PROCESS (Hayes, 2015).

## Results

Descriptive statistics and correlations between the study variables are displayed in Table 1.

**Table 1.** Descriptive Statistics and Correlations between Study Variables.

Variable	*M*	*SD*	1	2	3	4	5	6	7	8	9	10	11	12
Outcome variable														
1. Outcome events (goals)	1.26	1.20	–											
Control variables														
2. Place of game^a^	.50	–	.17***	–										
3. Team standing	.44	.19	.11***	.06**	–									
4. Opponent team standing	.44	.19	−.13***	−.06**	.16***	–								
5. Game number in season	19.5	10.97	.00	.00	.10***	.10***	–							
Predictor variables														
6. Loss disruption duration	2.42	10.66	−.05*	−.03	−.01	−.01	−.01	–						
7. Injury substitution disruption duration	13.04	23.75	−.03	.00	−.00	.03	.00	−.01	–					
8. Prior team familiarity	15.60	6.76	.16**	.06**	.32***	.00	−.22***	−.03	−.02	–				
9. Prior team familiarity squared	289.21	276.43	.14***	.04*	.28***	−.00	−.19***	−.02	−.01	.96***	–			
10. Current team familiarity	2.66	1.31	.04*	.01	.18***	.11***	.89***	−.03	−.03	−.01	−.02	–		
11. Current team familiarity squared	71.87	8.05	.05*	.01	.17***	.08***	.84***	−.03	−.02	.03	.02	.97***	–	
12. Team coordination	71.87	8.05	.19***	.07***	.27***	−.01	−.06***	−.05*	−.04	.26***	.27***	.00	.03	–

*Note*. *N* = 2280.

^a^0 = Away, 1 = Home.

*p < .05; **p < .01; ***p < .001.

In Table 2, the results for the prediction of team outcome events are displayed. In Model 1, we regressed team outcome events on the control variables season, place of game, game number in season, team’s standing, and opponent’s standing. We found that the place of game (*γ* = .35, *p* < .001) and the team’s standing (*γ* = .38, *p* = .015) had a significant positive effect on team outcome events, while the opponent’s standing (*γ* = −.82, *p* < .001) had a significant negative effect on team outcome events. In Model 2, we added loss disruption duration and injury substitution disruption duration. In support of Hypothesis 1a, we found that loss disruption duration had a significant negative effect on team outcome events (*γ* = −.01, *p* = .02). Against our assumptions, injury substitution disruption duration did not have a significant effect on team outcome events, thus we did not find evidence to support Hypothesis 1b. Accordingly, in line with Hypothesis 3a regarding outcome events, we found that loss disruption duration had a more severe effect on team outcome events than injury substitution disruption duration.

**Table 2.** Hierarchical Linear Modeling Results for Team Outcome Events (Goals).

	Team Outcome Events (Goals)					
	Model 1		Model 2 (H1a, H1b, H3a)		Model 3 (H4)	
	*γ* (SD)	*p*	*γ* (SD)	*p*	*γ* (SD)	*p*
Control variables						
Season dummies included	Yes		Yes		Yes	
Place of game^a^	.35*** (.04)	<.001	.35*** (.05)	<.001	.33*** (.05)	<.001
Game number in season	.00 (.00)	.635	.00 (.00)	.660	.00 (.00)	.326
Team standing	.38** (.16)	.015	.39** (.16)	.012	.32* (.15)	.035
Opponent team standing	−.82*** (.13)	<.001	−.82*** (.13)	<.001	−.80*** (.13)	<.001
Predictors						
Loss disruption duration			−.01* (.01)	.021	−.01* (.01)	.050
Injury substitution disruption duration			−.00 (.00)	.280	−.00 (.00)	.378
Team coordination					.02*** (.01)	<.001
τ (intercept)	1.22		1.25		−.48	
_σ_^2^	.11		.11		.27	
R^2^_marginal_/R^2^_conditional_	.04/.07		.05/.07		.07/.09	
*N* (level 1)	2280		2280		2280	
*N* (level 2a, games)	1140		1140		1140	
*N* (level 2b, teams	26		26		26	
AIC	7207.79		7227.53		7197.82	
BIC	7265.11		7296.31		7272.34	
Log-likelihood	−3593.90		−3601.76		−3585.91	

*Note*. *p*-values are based on two-sided tests.

*p < .05; **p < .01; ***p < .001.

^a^0 = Away, 1 = Home.

Table 3 displays the effects of team composition disruptions and prior team familiarity on team coordination. In Model 1, we regressed team coordination on the control variables. While place of game (*γ* = .66 *p* = .01) and team’s standing (*γ* = 3.54, *p* < .001) had a positive effect on team coordination, game number in season had a negative effect (*γ* = −.05, *p* < .001). The opponent’s standing had no effect on team coordination. In Model 2, we added the variables loss disruption duration and injury substitution disruption duration. In line with Hypothesis 2a, we found a negative effect of loss disruption duration on team coordination (*γ* = −.03, *p* = .01), hence the longer teams had to deal with this type of disruptive situation, the worse their coordination. We also found the hypothesized significant negative relationship between injury substitution disruption duration and team coordination (*γ* = −.01, *p* = .004), therefore providing support for Hypothesis 2b. However, this result should be interpreted with caution because there is no correlation between injury substitution disruption and team coordination (Table 1) and the relationship becomes only significant in the regression model using control variables. Therefore, in line with Hypothesis 3b, we found that loss disruption duration had a more severe effect on team coordination than injury substitution disruption duration.

**Table 3.** Hierarchical Linear Modeling Results: The Effects of Team Composition Disruptions and Prior Team Familiarity on Team Coordination.

	Team Coordination									
	Model 1		Model 2 (H2a, H2b, H3b)			Model 3 (H5)			Model 4 (H6a, H6b)	
	*γ* (SD)	*p*	*γ* (SD)		p	*γ* (SD)		*p*	*γ* (SD)	*p*
Control variables										
Season dummies included	Yes		Yes			Yes			Yes	
Place of game^a^	.66* (.27)	.013	.65* (.27)		.02	.63* (.27)		.019	.66** (.26)	.013
Game number in season	−.05*** (.01)	>.001	−.05*** (.01)		<.001	−.05 (.01)		<.001	−.05*** (.01)	<.001
Team standing	3.54*** (.92)	>.001	3.65*** (.92)		<.001	3.38*** (.93)		<.001	3.68*** (.92)	<.001
Opponent team standing	−.64 (.70)	.365	−.65 (.70)		.36	−.65 (.70)		.353	−.70 (.71)	.321
Predictors										
Loss disruption duration			−.03** (.01)		.005	−.03** (.01)		.005	−.09** (.03)	.002
Injury substitution disruption duration			−.01** (.01)		.004	−.01* (.01)		.047	−.01 (.01)	.577
Prior team familiarity			.01 (.04)		.78	.20 (.11)		.067	.01 (.04)	.886
Prior team familiarity X prior team familiarity						.00 (.00)		.061		
Interactions										
Loss disruption duration X prior team familiarity									.01* (.01)	.036
Injury substitution disruption duration X prior team familiarity									−.00 (.00)	.790
τ (intercept)	72.80		72.86			71.33			72.90	
_σ_^2^	1.06		1.23			1.47			1.23	
R^2^_marginal_/R^2^_conditional_	.09/.42		.09/.42			.10/.42			.10/.42	
*N* (level 1)	2280		2280			2280			2280	
*N* (level 2a, games)	1140		1140			1140			1140	
*N* (level 2b, teams)	26		26			26			26	
AIC	14924.98		14939.36			14966.49			14961.79	
BIC	14982.30		15013.87			15046.74			15047.77	
Log-likelihood	−7452.49		−7456.68			−7469.24			−7465.89	

*Note*. *p*-values are based on two-sided tests.

*p < .05; **p < .01; ***p < .001.

^a^0 = Away, 1 = Home.

Next, we added team coordination as a predictor of team outcome events in Table 2 Model 3. In line with our assumption, we found that team coordination had a positive effect on team outcome events (*γ* = .02, *p* < .001), thus we found support for Hypothesis 4.

**Figure 2.**
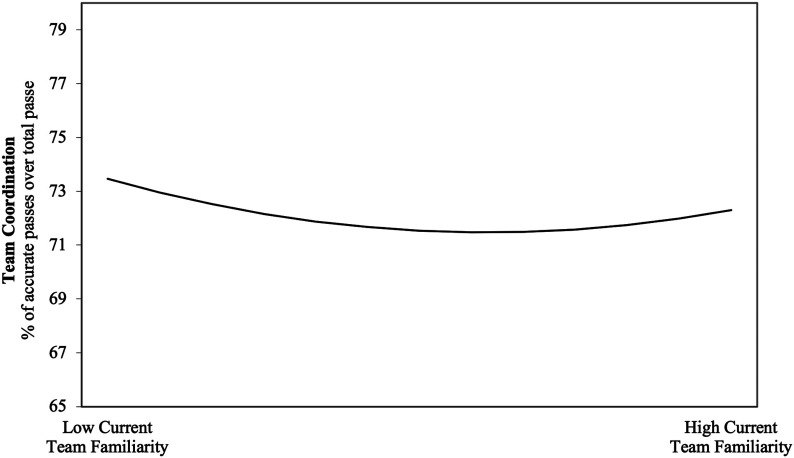
Reversing curvilinear effect of current team familiarity on team coordination.

Hypothesis 5 predicted an inverted U-shaped effect of team familiarity on team coordination. First, we tested the hypothesis for prior team familiarity. In Model 3 of Table 3, we added the squared term of prior team familiarity as an additional predictor of team coordination. There was no U-shaped relationship between prior team familiarity and team coordination. Second, we tested the hypothesis for current team familiarity. Table 4 displays the effects of team composition disruptions and current team familiarity on team coordination. In Model 3 of Table 4, we added the squared term of current team familiarity as a predictor of team coordination. The results indicated that current team familiarity and team coordination had a reversing (e.g., Gardner et al., 2017) or more precisely, a U-shaped relationship (*γ* = .35, *p* < .001); indicating that having established either a little or a lot of current team familiarity, within one EPL season, is better for team coordination than a moderate level of current team familiarity (see Figure 2). Thus, Hypothesis 5 was not supported for either prior or current team familiarity. That said, current team familiarity exhibited a curvilinear relationship with team coordination but not in the manner hypothesized.

**Table 4.** Hierarchical Linear Modeling Results: The Effects of Team Composition Disruptions and Current Team Familiarity on Team Coordination.

	Team Coordination							
	Model 1		Model 2		Model 3 (H5)		Model 4 (H6a, H6b)	
	γ (SD)	*p*	γ (SD)	*p*	γ (SD)	*p*	γ (SD)	*p*
Control variables								
Season dummies included	Yes		Yes		Yes		Yes	
Place of game^a^	.66* (.27)	.013	.65* (.27)	.015	.67* (.27)	.011	.67* (.27)	.013
Game number in season	−.05*** (.01)	>.001	−.05 (.03)	.084	−.02 (.03)	.042	−.05 (.03)	.099
Team standing	3.54*** (.92)	>.001	3.64*** (.92)	<.001	4.14*** (.93)	<.001	3.71*** (.92)	<.001
Opponent team standing	−.64 (.70)	.365	−.64 (.70)	.364	−.36 (.70)	.610	−.56 (.71)	.426
Predictors								
Loss disruption duration			−.03** (.01)	.004	−.03** (.01)	.006	−.04 (.03)	.160
Injury substitution disruption duration			−.01* (.01)	.044	−.01* (.01)	.032	−.03* (.01)	.014
Current team familiarity			.01 (.27)	.974	−2.16*** (.51)	<.001	−.11* (.28)	.679
Current team familiarity X current team familiarity					.35*** (.07)	<.001		
Interactions								
Loss disruption duration X current team familiarity							.00 (.01)	.882
Injury substitution disruption duration X current team familiarity							.01 (.00)	.080
τ (intercept)	72.80		73.02		74.83		73.22	
_σ_^2^	1.06		1.07		1.12		1.10	
R^2^_marginal_/R^2^_conditional_	.09/.42		.09/.42		.10/.42		.10/.42	
*N* (Level 1)	2280		2280		2280		2280	
*N* (Level 2a, games)	1140		1140		1140		1140	
*N (*Level 2b, teams)	26		26		26		26	
AIC	14924.98		14935.67		14916.66		14953.15	
BIC	14982.30		15010.18		14996.91		15039.13	
Log-likelihood	−7452.49		−7454.83		−7444.33		−7461.57	

*p*-values are based on two-sided tests.

*p < .05; **p < .01; ***p < .001.

^a^0 = Away, 1 = Home.

**Figure 3.**
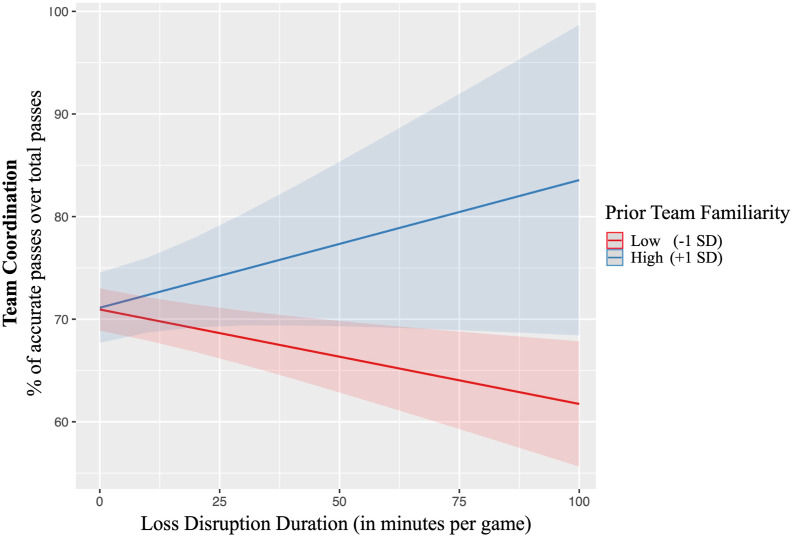
Moderating effect of prior team familiarity on the relationship between loss disruption duration and team coordination.

Next, we tested our moderation hypotheses. First, we tested Hypotheses 6a and 6b for prior team familiarity (see Table 3 Model 4). In support of Hypothesis 6a, we found a significant interaction between loss disruption duration and prior team familiarity (*γ* = .01, *p* = .036). Figure 3 shows that prior team familiarity mitigates the negative effect of loss disruption duration on team coordination. It seems that teams with high prior team familiarity might even be able to increase coordination as a reaction to disruption, whereas teams with low prior team familiarity show a negative effect between loss disruption duration and coordination (Figure 3).

Further, as seen in Table 3 Model 4, we did not find a significant interaction effect between injury substitution disruption duration and prior team familiarity on team coordination, thus we could not support Hypothesis 6b for prior team familiarity.

Results for Hypotheses 6a and b for current team familiarity are displayed in Table 4 Model 4. We did not find a significant interaction between loss disruption duration and current team familiarity nor between injury substitution disruption duration and current team familiarity. Thus, the analysis revealed that current team familiarity did not help buffer the negative effect of loss disruption or injury substitution on coordination.

In summary, we found that Hypothesis 6a was supported for prior team familiarity but not for current team familiarity, while Hypothesis 6b was not supported for prior as well as current team familiarity.

Regarding Hypothesis 7, we predicted that team familiarity moderates the indirect effects of (a) loss disruption duration on team outcome events and (b) injury substitution disruption duration on team outcome events through team coordination. First, we tested Hypothesis 7a and 7b for prior team familiarity. Following Hayes (2015), we calculated the index of moderated mediation and found that this index was significant (Estimate = .0001, 95% CI [.0001, .0003]). Thus, prior team familiarity moderated the indirect effect of loss disruption duration on team outcome events through team coordination. Table 5 displays the conditional indirect effects at varying levels of prior team familiarity. The indirect effect of loss disruption duration through team coordination on team outcome events was significant only for teams with low prior team familiarity (*b* = −.0013, *SE* = .0005, 95% CI [−.0024, −.0004]) and medium prior team familiarity (*b* = −.0007, *SE* = .0004, 95% CI [−.0015, −.0001]) and vanished for teams with high levels of prior team familiarity (*b* = .0002, *SE* = .0005, 95% CI [−.0009, .0012]). However, we did not find that prior team familiarity moderated the indirect effect of injury substitution disruption duration on team outcome events through team coordination (Estimate = .0000, 95% CI [.0000, .0000]).

**Table 5.** Summary of the Conditional Indirect Effects.

Conditional Indirect Effects	Level of Moderator	Estimate	*SE*	95% CI
Loss disruption duration → team coordination → team outcome event	Low prior team familiarity	−.0013	.0005	[−.0024, −.0004]
	Medium prior team familiarity	−.0007	.0004	[−.0013, −.0001]
	High prior team familiarity	.0002	.0005	[−.0009, .0012]

*Note.* Levels of the moderator are the mean +/− 1 *SD* from the mean. Confidence intervals for the indirect effects are based on 10,000 bootstrap samples.

Finally, we tested Hypothesis 7a and 7b for current team familiarity. The results revealed that current team familiarity did not moderate the indirect effect of loss disruption duration (Estimate = .00005, 95% CI [−.0005, .0006]) and injury substitution disruption duration (Estimate = .00015, 95% CI [−.0001, .0004]) on team outcome events through team coordination.

In summary, we found that Hypothesis 7a was supported for prior team familiarity but not for current team familiarity, while Hypothesis 7b was rejected for prior team familiarity as well as current team familiarity. Accordingly, our analysis addresses our Research Question in that current team familiarity appears most salient in shaping team coordination, but prior team familiarity appears to be most salient when examining team familiarity’s moderating influence on the relationship between team member loss and coordination and the indirect effect of team member loss on team outcome events through coordination.

## Discussion

We investigated the effects of two different forms of team composition disruptions (i.e., loss and injury substitution of a team member) that take place in the midst of the team’s task execution to understand the impact that they have on team coordination and team outcome events during time-limited performance episodes. As such, our research is most applicable to teams with set performance episodes. We additionally examined whether the loss of a team member was more severe than the substitution of a team member due to an injury in terms of their relationships with team coordination and outcome events. Simultaneously, we introduced team familiarity as a contextual determinant and stabilizing feature that can help teams to adapt and overcome team composition disruptions and thereby help them to keep up their coordination and performance and regain stability. Finally, we investigated whether differentiating between prior team familiarity and current team familiarity altered its effect on team coordination and more importantly on the relationship between team composition disruptions and team coordination.

Our analysis revealed that the duration that a team has to deal with the consequences of a disruption in the form of a team member loss negatively affected team outcome events as well as team coordination. Thus, the longer the team had to work without the dismissed team member, the worse their performance and coordination was. This finding is in line with previous studies that have investigated the effects of changing team membership on team coordination and of turnover and downsizing in organizations and teams on performance (e.g., DeRue et al., 2008; Summers et al., 2012; Van der Vegt et al., 2010). While we did not find a main effect relationship between the team composition disruption of substituting a team member and team outcome events, we did find evidence of the substitution form of composition disruption negatively impacting team coordination. In part, the lack of support for many of the substitution composition disruption relationships could be because the teams studied here (i.e., professional soccer teams) are composed of exceptional athletes and the gap in abilities between a starter and a substitute is not that extreme. Likewise, professional soccer teams often encounter the types of team composition disruptions examined here (a reason we studied this phenomenon in this context). However, this prevalence of such disruptions may result in the impact that they have on team outcome events to be smaller than in other contexts and therefore the relationships noted here may be more conservative than within other contexts. Furthermore, we found that team coordination affects team outcome events positively, which accords with previous team coordination and performance research (e.g., Fiore et al., 2001; Okhuysen & Bechky, 2009).

Moreover, we found differing effects of the two forms of team familiarity studied here (i.e., prior team familiarity and current team familiarity). Namely, we found that current team familiarity exhibited a reversing curvilinear relationship with coordination whereby at lower and higher levels of current team familiarity, team coordination was more positively impacted. However, at moderate levels of current team familiarity, team coordination was slightly lower. In contrast, prior team familiarity did not exhibit a significant relationship with team coordination. Accordingly, it appears more salient in terms of the impact on within-task coordination that the team has had more shared experiences within the current season.

Interestingly, we found that prior team familiarity seems to be the more salient form of team familiarity in helping a team overcome team composition disruptions especially when a team is facing an unexpected loss of a team member. We say this because teams with low prior team familiarity experienced more negative effects of loss disruption duration on team coordination and in turn performed worse compared to teams in which prior team familiarity was high. We did not find this effect for current team familiarity. Thus, our results indicate that teams with members who established a high level of team familiarity over the course of their career—especially outside the current team—were better able to overcome team composition disruptions due to the loss of a team member by keeping up their coordination. In contrast, establishing team familiarity only within the current team and thereby within a limited time span (i.e., one season) did not help teams to cope with the disruption of losing a team member. Perhaps the absence of a significant moderating impact of current familiarity is because there just is not enough time in the current season to develop the necessary shared cognition and emergent states that develop from working together and therefore, we only see a significant moderation effect when considering familiarity that is gained between team members over the course of their career prior to the current season (i.e. prior team familiarity).

So far, research has shown that team familiarity generally helps teams to perform better (e.g., Huckman et al., 2009; Reagans et al., 2005). However, our findings suggest that it is crucial to take a more differentiated view on team familiarity. Namely, our results suggest that the relationship between team familiarity and team coordination differs depending on whether such familiarity was built over the course of interactions in prior teams and organizations or whether it was built with only a consideration of the current team. In part, the fact that we include both forms of team familiarity within the current study may partially explain why we did not find the inverted-U relationship hypothesized to exist between team familiarity and coordination.

### Theoretical Implications

Our study has four main implications for team research. First, we demonstrate the salience of considering within-task composition disruptions in the first place. Prior work has predominantly only considered team composition changes that have occurred either prior to task execution or within transition episodes between tasks (e.g., Argote et al., 1995; Arrow & McGrath, 1993). That said, our study is focused on team composition changes that occur in the midst of task execution within teams. We argue that this type of disruption is becoming more prevalent in practice and therefore understanding the impacts of such within-task disruptions on team dynamics and team outcome events is of increasing importance. Given the evidence provided here that such within-task disruptions are salient, we hope that our work is a springboard for additional considerations of other types of within-task disruptions.

Second, as suggested by Christian et al. (2017, p. 76), our results showed that the “origin and duration of an adaptive stimulus may determine the ways that teams can maximize performance.” We investigated the effects of two different forms of team composition disruptions (i.e., loss and injury substitution of a team member) that take place during the team’s task execution and explored how the duration of these disruptions affects team coordination and outcome events and how they differ regarding their severities as introduced within EST. According to our results, when teams had to cope longer with the loss of a team member, they experienced higher coordination losses and showed lower performance. In contrast, the duration of a team member substitution due to an injury had less detrimental effects on team coordination and did not seem to adversely impact team performance.

These findings indicate that these two distinct forms of team composition disruptions are in fact different in terms of their criticality and as a result, appear to evoke different structural changes within teams and vary in their severity regarding their effects on coordination and performance. As such, our findings point out that different team composition disruptions seem to pose different stability demands on the team and that the duration of these disruptions matters. Thereby, our results advance our understanding of how teams are required to adapt their coordination to different types of stimuli (Christian et al., 2017; Grote et al., 2018; Maynard et al., 2015). Moreover, our results highlight the importance of distinguishing between different dimensions of events such as urgency, duration, and criticality as outlined within EST (Morgeson & DeRue, 2006; Morgeson et al., 2015). Therefore, our results echo the sentiments expressed by Christian et al. (2017) and Maynard et al. (2015) who stated that research needs to “flesh out the effects of different types of (disruptions or) triggers.” Accordingly, our hope is that our results provide an impetus for more research examining how teams adapt to different types of triggers within a single study so that the organizational team literature gains a deeper understanding of the differential effects that emerge from different types of triggers.

Third, we more comprehensively examine Morgeson and colleagues’ (2015) theorizing around EST. Namely, they articulated that events are apt to influence features, behaviors, and other events within the team. In our sample, teams encounter a variety of events within a given game and these events shape other behaviors that need to be altered in reaction to an event and one event experienced by a team impacts other events experienced within a given task episode. Likewise, while not studied here, what happens within a given game (i.e., game events) can impact subsequent games and the events that occur within them, and these can spill over into the entire season results. Morgeson et al. (2015) label this sequencing of events over time as an event chain, and it also connects to the underlying logic within EST that events happen across levels of analysis and our study opens up this consideration to future researchers interested in looking at events occurring across levels of analysis and along an event chain.

Given that we looked at team composition events (two different types) and how they impacted team coordination (i.e., a behavior) as well as other events within a task episode (i.e. goals scored), we feel that this is a more elaborate consideration of EST than has been done previously. Additionally, given that we look at the impact that team familiarity gained not only from current team interactions but also interactions between members on prior teams, we extend the thinking of event chains beyond the focal team which was not discussed within EST and therefore is an extension of this work.

As introduced above, our final theoretical contribution in this study is that we disentangled the temporal nature of team familiarity and introduced the distinction between current and prior team familiarity. In so doing, we have provided a greater attention to temporality (e.g., Ballard et al., 2008; Harrison et al., 2003) than has previously been done within the team familiarity literature. Specifically, we distinguish these two types of familiarity by taking the time horizons over which team familiarity was developed into account. Simultaneously, this also allows us to consider the fact that team members share a wide variety of experiences in different team contexts. While prior team familiarity helped the team to cope with the loss of a team member and thereby regain stability, the teams could not benefit in the same way from current team familiarity. These results show that the time frame over which team familiarity is developed within a team as well as the variety of experiences shared over different team contexts do matter when it comes to adapting coordination and in turn keeping up performance to certain forms of team composition disruptions (i.e., loss of a team member).

These findings imply that the shared work experience team members established in prior teams and organizations can be carried over to the current team and help teams manage disruptive situations better. In part, this relationship may be because team members know their fellow team members and their typical behaviors to a greater extent. Furthermore, we could show that even though prior team familiarity is beneficial for team coordination and performance when teams need to cope with the loss of a team member, prior as well as current team familiarity take on different dynamics when it comes to the direct impact that they have on team coordination. As such, our findings suggest that prior studies that have only focused on current team familiarity may be missing part of the story and future research may want to revisit some of these findings by not just focusing on current team familiarity but also including consideration of prior team familiarity. By doing so, future research will be able to see if the results noted here which suggest that prior team familiarity is more salient in certain situations translates to other settings.

### Practical Implications

Our findings generate new insights in the dynamics of teams. While our sample was drawn from professional sports teams, we can envision that our findings are salient for certain types of teams outside of this context. Specifically, sports teams can be classified as action teams that consist of highly skilled people closely synchronized with each other under brief performance events that require extended training (Sundstrom et al., 2000). Other teams that work under similar circumstances with a high need for coordination are project teams in organizations that have to meet deadlines or medical teams. Because teams that share certain characteristics can be highly alike even though they work in different industries (Cannon-Bowers & Bowers, 2006), our results can inform these industries and increase awareness about the effects of team composition disruptions characterized by structural changes in teams and their influence on coordination and performance.

In particular, our study highlights the fact that when teams lose a member, it is important to replace that individual as soon as possible as not doing so seems to have a more deleterious effect on team coordination and performance. We say this because the composition disruption caused by a substitution did not have as significant negative effects on coordination and performance as did the loss of a member. This may be because with a substitution, the team is able to maintain their level of knowledge and skill distribution by substituting one team member with another relatively equivalent team member. Thereby, following EST (Morgeson et al., 2015), we suppose that the loss of a team member is a more critical event for the team and therefore requires a higher level of team adaptation. In contrast, when teams are forced to substitute a team member due to injury, teams are able to keep up their coordination and performance by redistributing their resources and roles adequately which helps the team conserve their resources (e.g., Halbesleben et al., 2014; Hobfoll, 1989).

More specifically, our findings imply that the loss of a team member is a more harmful form of team composition disruption than the substitution of a team member. Hence, team leaders but also managers of organizations in general should be aware of the downsides that the loss of a team member has on team coordination and performance. To counteract these detriments, based on our findings we would recommend the substitution of team members rather than trying to put up with a loss of a team member. Losing a team member results in the team losing important team and task specific knowledge that is required to be able to perform well (Christian et al., 2014). Replacing this team member with a new employee who can be considered to have equivalent knowledge and skill sets ensures that teams do not suddenly lack crucial resources required to coordinate well and complete their task successfully (Halbesleben et al., 2014). Likewise, our results speak to the need to try to maintain an organization’s workforce to the extent possible so that such team composition disruptions are not encountered more than necessary.

However, as the current labor shortages indicate, this can be a challenge for organizations, but based on our findings, every attempt should be made to make the team as whole as possible following the loss of a team member. Thereby, managers and teams can learn how to counteract potential negative effects of team composition disruptions by reacting adequately when for example faced with team member loss. Unfortunately, following such a practice has the potential to overload members who are serving as substitutes within a new team while still having to maintain their current job responsibilities (e.g., Jones et al., 2007). Future research should investigate if there is a period of time over which individuals can serve as substitutes without negative consequences such as overload and stress. Likewise, while not relevant within our study given the context of our setting, within many organizations, substitutes are likely to be members of multiple teams which has been shown to have impacts on the amount of time that individuals can dedicate to each team (e.g., Margolis, 2020).

As such, organizations should pay close attention to maintaining employee job satisfaction and team viability as much as possible in order to reduce the occurrences of team composition disruptions examined here. This can include offering increased personal development opportunities to employees in an attempt to retain them as long as possible within the organization. By reducing the likelihood that employees will quit their job unexpectedly, organizations avoid the high costs of turnover in general but also reduce the difficulties encountered when trying to replace a team member quickly with an adequately qualified substitute which can be increasingly difficult within the tight labor market many organizations find themselves in.

Moreover, this study extends our knowledge on team adaptation processes. We find team familiarity to be an important contextual determinant of successful adaptation to disruptive structural changes (i.e., loss of a team member). Because team familiarity can help teams to keep a high level of coordination and performance when they are faced with the loss of a team member, managers and team leaders should consider how familiar the team members are when they compose their teams. Particularly, when they have to deal with the loss of a team member that cannot be substituted, managers are advised to pay special attention to the team’s prior team familiarity. In this situation, it is especially beneficial to compose teams by considering whether team members have had prior experience working together on preceding projects and teams even if this experience was obtained within a previous organization or another context. Thinking one step ahead, our findings show that managers that are hiring new people for their team and aim at building a team that can adapt swiftly to team composition disruptions, should look out for team members within the network of current team members and try to acquire these members as they already share some amount of familiarity with current team members. Accordingly, leveraging HR practices that include employee referrals might be beneficial especially in the current tight job market as it provides a way to learn about potential new employees but it may also have the advantage of allowing team members to possess familiarity that is not just based on interactions within current teams and within the current organization but also derived from interactions in prior projects and different organizations as this form of familiarity (i.e., prior familiarity) seems particularly salient in helping a team overcome composition disruptions.

### Limitations and Directions for Future Research

Sports teams can be categorized as highly interdependent action teams (Ishak & Ballard, 2011; Sundstrom et al., 2000). We found evidence of the findings discussed within the current study from three seasons of the EPL. Initial extensions of this work could include trying to leverage other methodological approaches to understand the mechanisms by which the relationships evidenced here are occurring. For instance, researchers could leverage mixed methods by trying to embed themselves within particular EPL teams (if staying in this same context) to understand what actions taken by the team during their training sessions and time in between games are most salient in building team familiarity that can engender the effects noted here. Additionally, if researchers are able to gain more access to teams within this setting, they may be able to assess flux in coordination like Summers et al. (2012) and assess whether examining flux in coordination surfaces any new insights to what was provided by the current study.

A potential limitation of our methodological approach concerns the assumption of non-interdependence of our outcomes. We used coordination and goals per team and game. Therefore, strictly speaking, games are counted twice (i.e., once for each team). However, we see goals and coordination per team as team-level event outcomes and not a game-level outcome (i.e., win, lose). While of course, the event outcomes are influenced by the opponent team (therefore, we control for it in the regression model), in theory, both teams have the possibility to score goals independent of each other’s goal count. Nevertheless, we tried to assess the impact of conducting our analysis in this way by running the complete analysis using only the home games from each team and cutting our sample size in half. The results, in terms of our hypotheses remained the same which further strengthens our results. The complete dataset is available upon request from the authors.

Likewise, it might be interesting for future researchers to examine the relationships examined here in other contexts to understand how they may be similar and different in other types of teams. For instance, examining similar constructs and relationships as examined here in other teams facing similar time-limited performance events such as project teams in organizations or medical teams (e.g., Buengeler et al., 2021) could be a valuable extension of the current work. We say this because there are some specific factors that make the current sample unique. For example, because team members in our sample move around in a restrictive labor market (i.e., specific sports leagues) they might have more possibilities to develop team familiarity through higher frequencies of mutual collaborations. In contrast, our sample was helpful in that it allowed us to track their movement across teams and their experience working with others across time. It might be challenging to attain detailed information on team members, their interactions as well as their careers within other industries and organizations. While these features allowed us to investigate team interactions over longer periods of time using objective data, future research needs to test whether our results translate to teams outside of the sports context, for example in sectors in which team members face less restrictions in changing their jobs and employers. Additionally, our sample is unique in many respects from other settings as it involves two teams competing directly against one another and when one team succeeds, the other team does not. Likewise, our sample is based on the best of the best within their profession (i.e., professional athletes) and therefore future work will need to evaluate whether the findings discussed here translate to other teams and if so, in what contexts do our findings translate? Nevertheless, research has shown that sports teams can provide an adequate context to investigate certain research questions in economics, management and work and organizational psychology (e.g., Ertug & Castellucci, 2013) and that parallels can be drawn between sports teams and teams operating in organizations (Gentry et al., 2017; Katz, 2001).

The variance explained within our study was relatively low. This may be the result of the fact that we did not assess which player was removed from the game either through loss or substitution and for that matter, who the specific player was and who was the substitute. Within the current study, we were merely focused on whether the actual act of a member loss or substitution, in and of itself had significant effects on coordination and team outcome events. And while they did, future research may find it beneficial to dig into who the actual players were who left the team and who was the replacement in the case of substitution. Doing so may result in more pronounced levels of variance explained when considering situations where the position that was lost or replaced was in a strategic core position (e.g., Humphrey et al., 2009). We argue that this would be a different study than the one that we performed here but may provide some interesting insights and research questions. Namely, such a study could analyze the relative skills of the person leaving the team with the person who is serving as the replacement.

Additionally, given that our analyses were correlational, we cannot draw conclusions about causality. In line with our research model, our results might imply that team members who are more familiar with each other actually manage to coordinate and perform better when they have to deal with the loss of a team member. Yet, our study design does not allow us to rule out the reversed effect. It might be possible that teams that coordinate and perform well are kept together longer and thus have greater chances to get more familiar with one another. Berman et al. (2002), investigated the causal effect of tacit knowledge on team performance and found evidence for mutual causality. Thus, on the one hand high-performing teams can be assumed to be kept together, while low-performing teams are more often reassembled to be able to achieve better results and remain competitive. On the other hand, the authors could show that “the creation of a collectively held stock of tacit knowledge by keeping a team together translates into a higher number of victories” (Berman et al., 2002, p. 26). Thus, future studies should use longitudinal or experimental designs to investigate whether team familiarity actually leads to better team coordination or whether these two constructs mutually influence each other as suggested by Berman et al. (2002).

Our study considered team familiarity as the shared experience a team has working together (Espinosa et al., 2007; Reagans et al., 2005). However, some authors have proposed that familiarity should be considered as a multidimensional construct comprising professional and personal familiarity (e.g., Maynard et al., 2018). While professional familiarity captures knowledge about work-related aspects (e.g., skill sets of other team members), personal familiarity captures knowledge about personal aspects of other team members (e.g., personal life situation). Thus, knowing that team members were working on the same team for a certain time span does not disclose anything about the specific type of familiarity that team members have established. Future research therefore should further investigate the quality of team members’ relationships. This could also help in understanding the differential effects we found between prior and current team familiarity, assuming that the quality of the relationship could possibly be the reason why we find varying results when looking at prior and current team familiarity. Furthermore, we calculated team familiarity based on the number of months a player played with each of the other team members. We felt that the month time period made sense in the current sample given the number of practices and matches afforded the teams in the EPL during a given month. That said, future researchers may want to examine the impact of calculating team familiarity using other lengths of time – i.e., number of weeks, etc.

Additionally, future research should disentangle whether the effects noted within the current study related to prior team familiarity depend on whether such familiarity was gained from within the same organization or in different organizations. Within the current study, we could not tease apart this effect based on the nature of our data, but it might be interesting to disentangle whether it matters if prior familiarity was gained from working on prior projects within the current organization or from projects within a different organization. Future research could investigate these relationships. Simultaneously, future research could additionally take into consideration how transition phases affect the development of team familiarity within teams and how this in turn affects team processes.

Moreover, our sample and study context did not allow us to investigate individual level effects, and this was beyond the scope of this study. Hence, we did not investigate individual team member’s role familiarity or identify which members hold strategic core roles (Humphrey et al., 2009) because multiple team members hold the same role and multiple roles are equally important for the task. Nevertheless, these might also be important variables to further advance our understanding of the effects team familiarity has on teamwork. Thus, future research should investigate how individual team member’s role familiarity impacts team coordination as well as whether team familiarity is beneficial for team adaptation even when the disruptive member change involves a strategic core team member opposed to a non-strategic core team member.

## Conclusion

This study highlights the impact that two different team composition disruptions can have on team coordination and team outcome events and suggests that the loss of a team member is the more detrimental composition disruption. Additionally, our results show that prior versus current team familiarity relates differently to team coordination depending on the time horizon over which team members developed familiarity. We thereby extend the familiarity literature by examining different time horizons over which team members build up team familiarity. This is important as prior team familiarity seems to be the more impactful form of team familiarity in terms of its impact in developing a context within which teams can more effectively handle the loss of a team member unexpectedly. Accordingly, within this study, we take a first step toward understanding how different disruptions impact team dynamics and outcomes and how team familiarity can assist teams in overcoming the challenges of such disruptions.

## Supplemental Material

Supplemental Material - How Team Familiarity Mitigates Negative Consequences of Team Composition Disruptions: An Analysis of Premier League Teams Supplemental Material for How Team Familiarity Mitigates Negative Consequences of Team Composition Disruptions: An Analysis of Premier League Teams by Surabhi Pasarakonda, Travis Maynard, Jan B. Schmutz, Patrick Lüthold, and Gudela Grote in Group & Organization Management
