# Supplementary material for: How Team Familiarity Mitigates Negative Consequences of Team Composition Disruptions: An Analysis of Premier League Teams
Source: Group Organ Manag. 2023 Aug 3;50(3):840–95. doi: 10.1177/10596011231193176 (PMC11999323; doi:10.1177/10596011231193176)
Supplement: Supplemental Material - How Team Familiarity Mitigates Negative Consequences of Team Composition Disruptions: An Analysis of Premier League Teams [file sj-pdf-1-gom-10.1177_10596011231193176.pdf]

Supplemental material

Example calculation of team familiarity for fictional team. Familiarity is always calculated per game. Therefore, in each game each team receives a new adapted familiarity value.

Player	Months played for the team	Months played with the other players	Sum	Min. played in current game	Player's team familiarity divided by 100 for game X
Player 1	62	(48+48+48+32+32+32+32+18+18+18+6+6+6)	344	90	30960
Player 2	48	(48+48+48+32+32+32+32+18+18+18+6+6+6)	344	90	30960
Player 3	48	(48+48+48+32+32+32+32+18+18+18+6+6+6)	344	70	24080
Player 4	48	(48+48+48+32+32+32+32+18+18+18+6+6+6)	344	90	30960
Player 5	32	(32+32+32+32+32+32+32+18+18+18+6+6+6)	296	60	17760
Player 6	32	(32+32+32+32+32+32+32+18+18+18+6+6+6)	296	90	26640
Player 7	32	(32+32+32+32+32+32+32+18+18+18+6+6+6)	296	90	26640
Player 8	18	(18+18+18+18+18+18+18+18+18+18+6+6+6)	198	80	15840
Player 9	18	(18+18+18+18+18+18+18+18+18+18+6+6+6)	198	90	17820
Player 10	6	(6+6+6+6+6+6+6+6+6+6+6+6+6)	78	90	7020
Player 11	6	(6+6+6+6+6+6+6+6+6+6+6+6+6)	78	90	7020
Player 12 (substitute 1)	32	(32+32+32+32+32+32+32+18+18+18+6+6+6)	296	20	5920
Player 13 (substitute 2)	18	(18+18+18+18+18+18+18+18+18+18+6+6+6)	198	30	5940
Player 14 (substitute 3)	6	(6+6+6+6+6+6+6+6+6+6+6+6+6)	78	10	780
			Total team score:		248340

Team's average team familiarity score: Total Team Score / (nPlayers -1) = 248340/14 = **17738.57**
Scaled Team's average team familiarity score = 17738.57/100 = **177.39**

Note. We have chosen simple numbers to make the calculations easier to interpret.
